# Relationships among Food Group Intakes, Household Expenditure, and Education Attainment in a General Japanese Population: NIPPON DATA2010

**DOI:** 10.2188/jea.JE20170248

**Published:** 2018-03-05

**Authors:** Tomomi Nagahata, Mieko Nakamura, Toshiyuki Ojima, Imako Kondo, Toshiharu Ninomiya, Katsushi Yoshita, Yusuke Arai, Takayoshi Ohkubo, Keiko Murakami, Nobuo Nishi, Yoshitaka Murakami, Naoyuki Takashima, Nagako Okuda, Aya Kadota, Naoko Miyagawa, Keiko Kondo, Tomonori Okamura, Hirotsugu Ueshima, Akira Okayama, Katsuyuki Miura

**Affiliations:** 1 Department of Nutrition, School of Health and Nutrition, Tokaigakuen University, Aichi, Japan; 2 Department of Community Health and Preventive Medicine, Hamamatsu University School of Medicine, Shizuoka, Japan; 3 Department of Food and Nutritional Sciences, College of Bioscience and Biotechnology, Chubu University, Aichi, Japan; 4 Department of Epidemiology and Public Health, Graduate School of Medical Sciences, Kyushu University, Fukuoka, Japan; 5 Department of Food and Human Health Science, Osaka City University Graduate School of Human Life Science, Osaka, Japan; 6 Department of Nutrition, Chiba Prefectural University of Health Sciences, Chiba, Japan; 7 Department of Hygiene and Public Health, Teikyo University School of Medicine, Tokyo, Japan; 8 International Center for Nutrition and Information, National Institute of Health and Nutrition, National Institutes of Biomedical Innovation, Health and Nutrition, Tokyo, Japan; 9 Department of Medical Statistics, School of Medicine, Toho University, Tokyo, Japan; 10 Department of Public Health, Shiga University of Medical Science, Shiga, Japan; 11 Department of Health and Nutrition, University of Human Arts and Sciences, Saitama, Japan; 12 Center for Epidemiologic Research in Asia, Shiga University of Medical Science, Shiga, Japan; 13 Department of Preventive Medicine and Public Health, Keio University School of Medicine, Tokyo, Japan; 14 Research Institute of Strategy for Prevention, Tokyo, Japan

**Keywords:** diet, food group, socioeconomic status, equivalent household expenditure, education attainment

## Abstract

**Background:**

A lower socioeconomic status (SES) may be related to the intake of unhealthy food; however, this relationship has not been examined in detail. This study was undertaken to examine relationships among food group intakes and SES in a representative Japanese population.

**Methods:**

This was a cross-sectional study using the baseline data of NIPPON DATA2010, which is a prospective cohort study of the National Health and Nutrition Survey in Japan. A total of 2,898 participants were included in the baseline survey in 2010. The effects of age (<65 years and ≥65 years), equivalent household expenditure (EHE), and education attainment on food group intakes (gram per 1,000 kcal) were analyzed using a two-way analysis of variance.

**Results:**

When EHE was lower, cereal intake was higher in men and women. Among men, fish, milk, and alcohol intakes were reduced with lower EHE. Among women, vegetable intake was reduced with lower EHE. In men and women, cereal intake was higher with lower education attainment. In contrast, meat intake was reduced with lower education attainment.

**Conclusions:**

Lower SES was associated with a higher cereal intake and lower vegetable, fish, meat, and milk intakes in a representative Japanese population. Socioeconomic discrepancies need to be considered in order to promote healthier dietary habits.

## INTRODUCTION

Japanese life expectancy has markedly increased since World War II. This increase may be attributed to a number of factors, one of which is nutritional improvements. Nationwide school lunch programs were initiated in 1954, resulting in increased body weight and height among Japanese children.^1^ Furthermore, daily energy and protein intakes have increased in Japan together with rapid economic growth.^1^ Japan in now in an age of plenty; however, a nutritionally unbalanced diet, mainly comprised of fat, particularly animal fat, and less carbohydrates and vegetables, has emerged as an important public health issue.^1^ A nutritionally unbalanced diet causes high blood pressure and dyslipidemia, which ultimately lead to cardiovascular diseases.^2^^,^^3^

The relationship between socioeconomic status (SES) and health has been extensively examined. Several reviews reported that individuals with lower SES have a nutritionally unbalanced diet, such as a diet with low fruit and vegetable intakes.^4^^,^^5^ Moreover, individuals with lower SES have higher energy and fat intakes and lower fiber, vitamin (including vitamin C, folates, β-carotene, and vitamin D), and mineral (such as calcium and iron) intakes.^5^^,^^6^ Low SES subgroups are known to consume diets of lower monetary value: a lower amount of pulses, vegetables, fruits, fish, meat, and dairy products, and a higher amount of grain, eggs, and fats among Japanese adults.^7^ However, controversy remains regarding the relationships among SES and some food group intakes, such as meat^8^^–^^11^ and fish.^12^^–^^14^

According to national representative data from the National Diet and Nutrition Survey in the United Kingdom, higher SES (household income, occupational class, and educational attainment) groups have higher fruit and vegetable intakes, but lower red and processed meat and non-milk extrinsic sugar intakes.^15^ In Japan, Nishi et al reported that subjects with a lower household income consumed more cereals, but less vegetables, fruit, mushrooms, fish, meat, and milk products using the data from the National Health and Nutrition Survey (NHNS)^16^ in 2010 and 2011. Dietary data used in the present study were a part of the data analyzed by Nishi et al; however, the relationships among food group intakes, equivalent household expenditure (EHE), and education attainment have not yet been investigated. Therefore, the present study was undertaken to examine the characteristics of food group intakes in relation to SES, EHE, and educational attainment in a representative Japanese population.

## METHODS

### Study population

This was a cross-sectional study based on the National Integrated Project for Prospective Observation of Non-communicable Disease and its Trends in the Aged 2010 (NIPPON DATA2010). NIPPON DATA2010 is a prospective cohort study with the aim of preventing cardiovascular diseases in Japan. The baseline survey was performed at the same time as NHNS in 2010, which was conducted by the Ministry of Health, Labour and Welfare of Japan. Furthermore, these data were linked to the Comprehensive Survey of Living Conditions (CSLC) in June 2010. The details of NHNS2010,^17^^,^^18^ CSLC2010,^19^^,^^20^ and NIPPON DATA 2010^21^^–^^24^ have been described elsewhere.

Briefly, in November 2010, 8,815 residents aged 1 year and older from 300 randomly selected districts throughout Japan participated in the dietary survey for NHNS2010. Overall, 7,229 participants (3,334 men and 3,895 women) were older than 20 years. A total of 3,873 participants (1,598 men and 2,275 women) had a blood test in NHNS2010, and 2,898 (1,239 men and 1,659 women) agreed to participate in the baseline survey for NIPPON DATA2010. The age distributions of NHNS2010 and NIPPON DATA2010 have already been reported.^22^ The distribution of population by age and sex of NIPPON DATA2010 was similar to that of NHNS2010 with blood tests. In the present study, 2,891 participants (1,236 men and 1,655 women) merged with CSLC2010 were included.

Trained researchers or examiners obtained informed consent from participants before enrolling in the study.

### Measurements

Information on variables for dietary intakes was obtained from the dietary survey for NHNS2010. Dietary intakes were assessed using the “1-day household-based food weighing with approximated proportions method”, and the details of this method have been described elsewhere.^25^ In brief, this method was developed to estimate the food intake of individual subjects using a household-based dietary survey. The survey employed a method of approximated proportions of each dish or food shared by family members in the household. All foods were categorized into 98 small groups, 33 medium groups, and 17 large groups in NHNS. In this study, nine groups with relatively high intakes were analyzed among 17 large groups that had a median intake of more than 10 g: cereals, potatoes and starches (potatoes), soy and soy products including other pulses (soy), vegetables, fruit, fish and shellfish (fish), meat, eggs, and milk products (milk). Moreover, alcohol (including sake, beer, and western liquor) intake was analyzed. Food group intakes were adjusted for total energy intakes using the density method (grams per 1,000 kcal).

EHE, education attainment, and household income per year were surveyed using self-administered questionnaires for CSLC2010, NIPPON DATA2010, and NHNS2010. EHE per month was filled out as household expenses in May, excluding taxes, social security fees, business payments, savings, loans, and life insurances. Of the 2,891 participants, 84 with missing data (29 men and 55 women) and 139 with unknown data (filled out of 999; 51 men and 88 women) were excluded, resulting in 2,668 (1,156 men and 1,512 women) for the analysis. Regarding EHE, household expenses were divided by the square root of the number of household members. In the present study, EHE was reclassified into four categories by sex-specific quartiles (Q1–Q4).

Education attainment was selected from six categories: none, elementary school, junior high school, high school, college, and university. Of the 2,891 participants, 6 were missing data (5 men and 1 woman) and were excluded, resulting in 2,885 (1,231 men and 1,654 women) for the analysis. In the present study, education attainment was reclassified into three categories: ≤junior high school, high school, and college or university.

Household annual income was selected from four categories: <2 million yen, 2–6 million yen, ≥6 million yen, and “Don’t know”. Of the 2,891 participants, 173 were missing data (65 men and 108 women) and 122 had unknown data (filled out “Don’t know”; 46 men and 76 women) on household income and were excluded, resulting in 2,596 (1,125 men and 1,471 women) for the analysis on household income. Household income was adjusted by household size; the square root of the number of household members, instead of calculating individual household incomes.

### Statistical analysis

Age was divided into 2 groups: <65 years (younger) and ≥65 years (older). The effects of age, EHE, education attainment, and household income on food group intakes were analyzed using a two-way analysis of variance (ANOVA). Concurrently, the interactions between EHE or educational attainment or household income and age were also analyzed. Analyses were performed using IBM SPSS Statistics version 22.0 (Tokyo, Japan). All tests with a difference of *P* < 0.05 were considered to be significant.

### Ethical considerations

The NIPPON DATA2010 protocol was approved by the Institutional Review Board of Shiga University of Medical Science (No. 22-29, 2010).

## RESULTS

Table 1 shows the distribution of subjects by EHE quartile or education attainment in each age group by sex in 2010. The mean numbers of male household members were Q1: 3.14 (SD, 1.67), Q2: 2.99 (SD, 1.43), Q3: 2.98 (SD, 1.38), and Q4: 2.64 (SD, 1.42), while the mean numbers of female household members were Q1: 2.94 (SD, 1.61), Q2: 2.93 (SD, 1.41), Q3: 2.97 (SD, 1.42), and Q4: 2.61 (SD, 1.34).

**Table 1.** Number (%) of participants by equivalent household expenditure and education attainment: NIPPON DATA2010

	Age, years	Equivalent household expenditure^a^					Educational attainment^a^			
	
		Q1	Q2	Q3	Q4	Total	≤Junior high school	High school	College and University	Total
Men^b^	<65	167 (27.0)	167 (27.0)	145 (23.5)	139 (22.5)	618 (100.0)	77 (12.5)	281 (45.8)	256 (41.7)	614 (100.0)
	≥65	112 (21.8)	122 (23.8)	127 (24.8)	152 (29.6)	513 (100.0)	204 (39.8)	194 (37.9)	114 (22.3)	512 (100.0)
	Total	279 (24.7)	289 (25.6)	272 (24.0)	291 (25.7)	1131 (100.0)	281 (25.0)	475 (42.2)	370 (32.9)	1126 (100.0)

Women^c^	<65	198 (22.3)	229 (25.8)	224 (25.3)	236 (26.6)	887 (100.0)	95 (10.7)	400 (45.1)	391 (44.1)	886 (100.0)
	≥65	170 (28.1)	151 (25.0)	126 (20.8)	158 (26.1)	605 (100.0)	256 (42.3)	287 (47.4)	62 (10.2)	605 (100.0)
	Total	368 (24.7)	380 (25.5)	350 (23.5)	394 (26.4)	1492 (100.0)	351 (23.5)	687 (46.1)	453 (30.4)	1491 (100.0)

^a^*n* (%).

^b^Q1–Q4 show quartiles for men, Q1: ≤89.5, Q2: 89.6–132.9, Q3: 133.0–175.1, Q4: ≥175.2 (thousand Yen per month).

^c^Q1–Q4 show quartiles for women, Q1: ≤90.1, Q2: 90.2–135.1, Q3: 135.2–175.1, Q4: ≥175.2 (thousand Yen per month).

Table 2 shows the mean values of 10 food group intakes by the quartiles of EHE. Cereal intake correlated with EHE in men and women. Cereal intake was higher with lower EHE in men and women. Among men, fish, milk, and alcohol intakes were reduced with lower EHE. Among women, vegetable intake was reduced with lower EHE. On the other hand, many food group intakes correlated with age. Among men, younger participants (<65 years) consumed less soy, vegetables, fruit, fish, and milk than older participants (≥65 years). Among women, younger participants consumed less potatoes, soy, vegetables, fruit, and fish than older participants. In contrast, younger participants consumed more meat and alcohol than older participants in men and women.

**Table 2.** Relationships among food group intakes and equivalent household expenditure: NIPPON DATA2010

	Age, years	Men							Women						
	
		Equivalent household expenditure^a^^,^^f^						*P*^e^	Equivalent household expenditure^a^^,^^g^						*P*^e^
	
		Q1	Q2	Q3	Q4	Total			Q1	Q2	Q3	Q4	Total		
Cereals	<65	267 (81)^*d^	256 (76)^*d^	245 (71)	232 (66)	251 (75)	EHE	<0.001	235 (70)^*d^	219 (72)	231 (77)^*d^	204 (64)	222 (72)	EHE	<0.001
	≥65	256 (73)^*d^	256 (75)^*d^	242 (73)	221 (64)	242 (72)	Age	0.131	248 (72)^*c,d^	233 (69)^*d^	224 (69)	205 (58)	228 (69)	Age	0.151
	Total	262 (78)^*c,d^	256 (75)^*d^	244 (72)^*d^	226 (65)	247 (74)	Interaction	0.766	241 (71)^*b,d^	225 (71)^*d^	229 (74)^*d^	205 (62)	224 (71)	Interaction	0.128

Potatoes	<65	28 (32)	27 (29)	26 (32)	26 (32)	27 (31)	EHE	0.896	30 (31)	31 (39)	28 (36)	30 (38)	30 (36)	EHE	0.678
	≥65	30 (35)	30 (39)	30 (35)	29 (34)	30 (35)	Age	0.157	40 (44)	34 (39)	35 (37)	39 (37)	37 (40)	Age	0.001
	Total	29 (33)	29 (33)	28 (33)	28 (33)	28 (33)	Interaction	0.988	35 (38)	32 (39)	31 (37)	34 (38)	33 (38)	Interaction	0.641

Soy	<65	31 (40)	29 (44)	28 (38)	30 (33)	30 (39)	EHE	0.645	40 (45)^*b^	28 (36)	34 (41)	36 (43)	34 (41)	EHE	0.114
	≥65	40 (42)	37 (44)	39 (43)	33 (34)	37 (41)	Age	0.002	43 (44)	41 (44)	38 (35)	43 (43)	42 (42)	Age	0.002
	Total	35 (41)	32 (44)	33 (41)	31 (34)	33 (40)	Interaction	0.602	42 (45)	33 (40)	35 (39)	38 (43)	37 (42)	Interaction	0.443

Vegetables	<65	132 (77)	137 (86)	137 (79)	138 (77)	136 (80)	EHE	0.713	158 (93)	156 (95)	155 (90)	169 (91)	160 (93)	EHE	0.030
	≥65	159 (106)	166 (87)	161 (82)	168 (89)	164 (91)	Age	<0.001	182 (100)^*d^	192 (102)	198 (92)	213 (109)	196 (102)	Age	<0.001
	Total	143 (90)	149 (88)	148 (81)	154 (85)	148 (86)	Interaction	0.984	169 (97)^*d^	170 (99)	171 (93)	187 (101)	175 (98)	Interaction	0.464

Fruit	<65	28 (50)	34 (45)	43 (66)	43 (51)	37 (53)	EHE	0.078	59 (68)	72 (84)	64 (79)	67 (67)	66 (75)	EHE	0.791
	≥65	71 (69)	73 (60)	67 (62)	82 (68)	74 (65)	Age	<0.001	101 (80)	84 (75)	90 (77)	99 (72)	94 (76)	Age	<0.001
	Total	45 (62)	51 (55)	54 (65)	63 (63)	54 (62)	Interaction	0.249	79 (76)	77 (81)	73 (80)	79 (71)	77 (77)	Interaction	0.062

Fish	<65	37 (38)	34 (33)	42 (40)	43 (32)	39 (36)	EHE	0.015	40 (41)	38 (37)	40 (40)	44 (37)	41 (39)	EHE	0.079
	≥65	52 (44)	44 (32)	52 (39)	55 (40)	51 (39)	Age	<0.001	46 (39)	44 (51)	44 (34)	52 (35)	47 (40)	Age	0.004
	Total	43 (41)	38 (33)^*d^	47 (40)	49 (37)	44 (38)	Interaction	0.789	43 (40)	40 (43)	42 (38)	47 (36)	43 (39)	Interaction	0.893

Meat	<65	43 (36)	46 (32)	49 (37)	46 (35)	46 (35)	EHE	0.723	42 (35)	43 (32)	43 (34)	45 (37)	43 (34)	EHE	0.287
	≥65	30 (29)	29 (27)	28 (25)	32 (26)	30 (27)	Age	<0.001	28 (28)	31 (27)	34 (25)	32 (28)	31 (27)	Age	<0.001
	Total	38 (34)	39 (32)	39 (34)	39 (31)	39 (32)	Interaction	0.479	35 (32)	39 (30)	40 (32)	40 (34)	38 (32)	Interaction	0.946

Eggs	<65	20 (18)	17 (15)	18 (17)	17 (16)	18 (16)	EHE	0.440	20 (20)	21 (21)	18 (17)	20 (17)	20 (19)	EHE	0.589
	≥65	18 (16)	18 (17)	16 (17)	18 (16)	18 (17)	Age	0.894	18 (19)	21 (21)	22 (21)	19 (18)	20 (20)	Age	0.966
	Total	19 (17)	17 (16)	17 (17)	18 (16)	18 (17)	Interaction	0.685	19 (20)	21 (21)	19 (19)	19 (18)	20 (19)	Interaction	0.158

Milk	<65	30 (48)	40 (55)	37 (58)	45 (57)	38 (55)	EHE	<0.001	56 (71)	70 (72)	61 (72)	66 (75)	63 (73)	EHE	0.050
	≥65	35 (53)^*b,d^	57 (67)	50 (61)	69 (67)	54 (64)	Age	<0.001	58 (70)	70 (70)	62 (71)	72 (67)	66 (69)	Age	0.571
	Total	32 (50)^*b,d^	47 (61)	43 (60)	57 (64)	45 (59)	Interaction	0.257	57 (70)	70 (71)	62 (72)	68 (72)	64 (71)	Interaction	0.923

Alcohol	<65	80 (129)^*c^	77 (133)^*c^	158 (220)	111 (160)	105 (165)	EHE	<0.001	27 (97)	38 (115)	33 (93)	41 (101)	35 (102)	EHE	0.119
	≥65	71 (106)	90 (146)	87 (138)	100 (145)	88 (136)	Age	0.043	10 (39)	21 (70)	9 (34)	21 (72)	16 (57)	Age	<0.001
	Total	77 (120)^*c^	82 (138)^*c^	125 (189)	106 (152)	97 (153)	Interaction	0.007	19 (76)	31 (100)	24 (78)	33 (91)	27 (87)	Interaction	0.937

EHE, equivalent household expenditure.

^a^Means (SD) (g/1,000 kcal).

^*b^Multiple comparisons compared with Q2, *P* < 0.05.

^*c^Multiple comparisons compared with Q3, *P* < 0.05.

^*d^Multiple comparisons compared with Q4, *P* < 0.05.

^e^Main effect or interaction by ANOVA, adjusted by the type of house.

^f^Q1–Q4 show quartiles for men, Q1: ≤89.5, Q2: 89.6–132.9, Q3: 133.0–175.1, Q4: ≥175.2 (thousand Yen per month).

^g^Q1–Q4 show quartiles for women, Q1: ≤90.1, Q2: 90.2–135.1, Q3: 135.2–175.1, Q4: ≥175.2 (thousand Yen per month).

The intake of alcohol in men and fruit in women showed a significant interaction between EHE and age. Alcohol intake was reduced with lower EHE among younger men but not older men. Fruit intake was higher with lower EHE among older women but not younger women.

In comparisons with effects by household income, eTable 1 shows the distribution of subjects by household income in each age group by sex in 2010. eTable 2 shows relationships among food group intakes and household income. Cereal and meat intakes correlated with household income in men and women, whereas meat intake did not correlate with EHE.

Table 3 shows the relationships among food group intakes and education attainment. Cereal and meat intakes correlated with education attainment in men and women. Cereal intake was higher with lower education attainment. In contrast, meat intake was reduced with lower education attainment. Fish intake correlated with education attainment in women. Fish intake was higher with lower education attainment. On the other hand, many food group intakes correlated with age. The relationships among food group intakes and age were similar to those shown in Table 2.

**Table 3.** Relationships among food group intakes and education attainment: NIPPON DATA2010

	Age, years	Men						Women					
	
		Educational attainment^a^					*P*^d^	Educational attainment^a^					*P*^d^
	
		≤Junior high school	High school	College and University	Total			≤Junior high school	High school	College and University	Total		
Cereals	<65	269 (78)^*c^	253 (76)	245 (72)	251 (75)	Education attainment	<0.001	234 (72)	218 (72)	223 (72)	222 (72)	Education attainment	<0.001
	≥65	254 (76)^*c^	242 (66)^*c^	219 (69)	242 (72)	Age	<0.001	246 (71)^*b,c^	220 (63)^*c^	193 (69)	228 (69)	Age	0.243
	Total	258 (76)^*b,c^	248 (72)^*c^	237 (72)	247 (74)	Interaction	0.392	243 (72)^*b,c^	219 (68)	219 (72)	224 (71)	Interaction	0.002

Potatoes	<65	28 (31)	26 (30)	27 (32)	27 (31)	Education attainment	0.376	32 (40)	32 (38)	28 (34)	30 (36)	Education attainment	0.416
	≥65	32 (38)	27 (31)	31 (38)	30 (35)	Age	0.183	38 (42)	37 (38)	34 (35)	37 (40)	Age	0.018
	Total	31 (36)	26 (30)	28 (34)	28 (33)	Interaction	0.755	36 (42)	34 (38)	29 (34)	33 (38)	Interaction	0.984

Soy	<65	37 (43)	27 (35)	30 (42)	29 (39)	Education attainment	0.097	34 (41)	34 (39)	35 (44)	34 (41)	Education attainment	0.718
	≥65	39 (42)	35 (37)	37 (43)	37 (41)	Age	0.031	39 (44)	43 (41)	45 (40)	42 (42)	Age	0.003
	Total	38 (42)	30 (36)	32 (42)	33 (40)	Interaction	0.706	38 (43)	38 (40)	36 (44)	37 (42)	Interaction	0.818

Vegetables	<65	140 (96)	132 (77)	139 (78)	136 (80)	Education attainment	0.073	158 (96)	160 (91)	160 (93)	160 (93)	Education attainment	0.264
	≥65	163 (90)	157 (92)	178 (87)	164 (91)	Age	<0.001	185 (101)^*b^	206 (101)	197 (102)	196 (102)	Age	<0.001
	Total	157 (92)	142 (84)	151 (83)	148 (86)	Interaction	0.448	178 (101)	179 (98)	165 (95)	175 (98)	Interaction	0.336

Fruit	<65	44 (53)	35 (54)	37 (53)	37 (53)	Education attainment	0.112	81 (95)	62 (67)	66 (78)	66 (75)	Education attainment	0.931
	≥65	67 (66)^*c^	73 (62)	88 (67)	74 (65)	Age	<0.001	86 (77)	101 (78)	98 (63)	94 (76)	Age	<0.001
	Total	61 (63)	50 (60)	53 (63)	54 (62)	Interaction	0.018	84 (82)	78 (74)	71 (77)	77 (77)	Interaction	0.007

Fish	<65	41 (37)	40 (37)	38 (35)	39 (36)	Education attainment	0.511	52 (41)^*c^	43 (40)^*c^	36 (36)	41 (39)	Education attainment	0.018
	≥65	51 (40)	52 (39)	48 (35)	51 (39)	Age	<0.001	48 (46)	46 (36)	44 (31)	47 (40)	Age	0.302
	Total	49 (40)	45 (38)	41 (35)	44 (38)	Interaction	0.939	49 (45)^*c^	44 (38)	37 (35)	43 (39)	Interaction	0.235

Meat	<65	33 (27)^*b,c^	46 (36)	50 (35)	46 (35)	Education attainment	0.001	30 (29)^*b,c^	44 (34)	47 (36)	43 (35)	Education attainment	<0.001
	≥65	28 (27)	32 (27)	31 (25)	30 (27)	Age	<0.001	26 (27)^*b^	35 (27)	35 (26)	31 (27)	Age	<0.001
	Total	29 (27)^*b,c^	40 (33)	44 (33)	39 (32)	Interaction	0.046	27 (28)^*b,c^	40 (31)	45 (35)	38 (32)	Interaction	0.461

Eggs	<65	19 (20)	18 (16)	18 (15)	18 (16)	Education attainment	0.744	22 (22)	20 (19)	19 (18)	20 (19)	Education attainment	0.552
	≥65	18 (17)	17 (17)	18 (16)	18 (17)	Age	0.731	20 (22)	20 (18)	19 (17)	20 (20)	Age	0.529
	Total	18 (18)	18 (16)	18 (16)	18 (17)	Interaction	0.876	20 (22)	20 (18)	19 (18)	20 (19)	Interaction	0.848

Milk	<65	33 (54)	36 (54)	41 (56)	38 (55)	Education attainment	0.088	62 (76)	63 (74)	65 (71)	64 (73)	Education attainment	0.175
	≥65	45 (63)^*b^	61 (66)	58 (58)	54 (64)	Age	<0.001	56 (67)^*b^	73 (71)	72 (67)	66 (69)	Age	0.415
	Total	42 (61)	47 (60)	46 (57)	45 (59)	Interaction	0.376	57 (69)	67 (73)	66 (70)	64 (71)	Interaction	0.289

Alcohol	<65	109 (182)	111 (165)	96 (161)	105 (165)	Education attainment	0.772	42 (173)	34 (91)	34 (89)	35 (102)	Education attainment	0.813
	≥65	96 (140)	76 (123)	96 (147)	88 (136)	Age	0.106	15 (53)	15 (59)	18 (63)	16 (57)	Age	<0.001
	Total	99 (153)	97 (150)	96 (156)	97 (153)	Interaction	0.270	23 (101)	26 (80)	32 (86)	27 (87)	Interaction	0.753

^a^Means (SD) (g/1,000 kcal).

^*b^Multiple comparisons compared with High school, *P* < 0.05.

^*c^Multiple comparisons compared with College and University, *P* < 0.05.

^d^Main effect or interaction by ANOVA.

Some food group intakes—fruit and meat in men and fruit and cereals in women—exhibited a significant interaction between education attainment and age. In younger men and women, fruit intake was higher with lower education attainment. In contrast, in older men and women, fruit intake was reduced with lower education attainment.

Since EHE and education attainment may differ by location, the analyses of this study were also conducted by adjusting for area blocks; however, this did not markedly affect the results obtained.

## DISCUSSION

In this study on a representative Japanese population, we examined the intakes of nine food groups and alcohol in relation to EHE and education attainment. Cereal intake was higher with lower EHE in men and women. Furthermore, milk intake in men and vegetable intake in women were reduced with lower EHE. Cereal intake was higher and meat intake was reduced with lower education attainment in men and women.

Nishi et al reported that cereal intake was higher, while vegetable, fruit, mushroom, fish and shellfish, meat, and milk product intakes were lower in the low-household-income group.^16^ In the present study, cereal intake was also higher with lower EHE, while meat intake was reduced with lower educational attainment. To the best of our knowledge, this is the first study to examine food group intakes by education attainment in a general Japanese population, except for a study of Japanese women during pregnancy.^26^ According to a review conducted by Darmon and Drewnowski,^5^ the intake of grains and starchy vegetables (including bread, pasta, rice, and cereals) was high among low-SES individuals. Our results on cereal intake were consistent with these findings. In Japan, lower neighborhood SES has been associated with a higher cereal intake.^16^ Moreover, in this study, meat intake was reduced with low education attainment and household income (eTable 2), which is consistent with previous findings from Western countries^8^^,^^9^ and Japan.^7^^,^^16^ However, contrasting findings have also been reported.^11^^,^^12^ Since dietary habits are affected by population groups, religion, and residence, the relationships among meat intake and SES warrant further study.

In this study, meat intake was reduced with a lower household income but not lower EHE. Higher EHE has been associated with a balanced nutritional intake in Japanese adults.^27^ However, this is the first study on food group intakes by EHE. In this study, EHE was reduced with a lower household income in this population (data not shown); however, some of this population had higher EHE due to savings in part from the elderly with a lower household income. Therefore, the population with a higher household income may differ from that with higher EHE.

In this study, fruit intake exhibited a significant interaction between education attainment and age. Among elderly men and women, fruit intake was lower in participants with less than junior high school education than those more education. Correspondingly, a previous study reported that women obtaining higher education qualifications consumed more fruit and vegetables.^28^ Nakamura et al found that a higher education correlated with a greater vegetable intake using information on nutrition labels and conversations with family or friends during meals in Japanese adults.^29^ Based on these findings, highly educated individuals appear to have learned more about healthy food habits in the school period, resulting in higher vegetable and fruit intakes. Among younger men and women, however, fruit intake was higher in participants with less than junior high school education than those more education, and we did not take this into consideration as a confounding factor. Different factors between younger and older individuals may mediate fruit intake.

There are two main limitations in this study. The design of this study was cross-sectional. Therefore, our data do not explain any causal relationships among SES and food group intakes. Furthermore, the dietary intake survey for NHNS was performed on 1 day in November. Therefore, marketing variance by season may affect food group intakes in this study.

Beyond these limitations, it is important that cereal intake was higher with lower EHE and educational attainment. Moreover, meat intake was reduced among those with lower educational attainment using the national survey in Japan. Socioeconomic discrepancies need to be considered in order to promote healthier dietary habits.

## APPENDIX A. SUPPLEMENTARY DATA

eTable 1. Number (%) of participants by household income: NIPPON DATA2010

eTable 2. Relationships among food intakes and household income: NIPPON DATA2010
