# Supplementary material for: Relationships among Food Group Intakes, Household Expenditure, and Education Attainment in a General Japanese Population: NIPPON DATA2010
Source: J Epidemiol. 2018 Mar 5;28(Suppl 3):S23–8. doi: 10.2188/jea.JE20170248 (PMC5825688; doi:10.2188/jea.JE20170248)
Supplement: Supplementary file 1 [file je-28-S023-s001.pdf]

eTable 1. Number (%) of participants by household income: NIPPON DATA2010

	Age, years	Household income							
		<2 million Yen		2–6 million Yen		≥6 million Yen		Total	
Men	<65	89	(14.9)	344	(57.6)	164	(27.5)	597	(100.0)
	≥65	119	(23.6)	319	(63.3)	66	(13.1)	504	(100.0)
	Total	208	(18.9)	663	(60.2)	230	(20.9)	1101	(100.0)
Women	<65	118	(13.6)	503	(57.9)	248	(28.5)	869	(100.0)
	≥65	205	(34.9)	328	(55.8)	55	(9.4)	588	(100.0)
	Total	323	(22.2)	831	(57.0)	303	(20.8)	1457	(100.0)

n (%)

eTable 2. Relationships among food intakes and household income: NIPPON DATA2010

	Age, years	Men: Household income[a] <2 million yen	Men: Household income[a] 2–6 million yen	Men: Household income[a] ≥6 million yen	Men: Total		Men: p [d]	Women: Household income[a] <2 million yen	Women: Household income[a] 2–6 million yen	Women: Household income[a] ≥6 million yen	Women: Total		Women: p [d]
Cereals	<65	261 (83) [*c]	250 (72)	243 (73)	250 (74)	Household income	<0.001	221 (65)	225 (77) [*c]	213 (63)	221 (72)	Household income	<0.001
	≥65	262 (81) [*b,c]	238 (65)	235 (72)	243 (71)	Age	0.586	237 (71) [*b,c]	227 (66) [*c]	209 (64)	229 (68)	Age	0.018
	Total	261 (82) [*b,c]	244 (69)	241 (73)	247 (73)	Interaction	0.476	231 (70) [*c]	225 (73) [*c]	212 (63)	224 (70)	Interaction	0.031
Potatoes	<65	25 (30)	26 (31)	26 (31)	26 (30)	Household income	0.987	31 (38)	30 (36)	28 (34)	29 (36)	Household income	0.088
	≥65	28 (36)	30 (35)	31 (32)	30 (35)	Age	0.032	41 (47) [*b]	34 (35)	33 (38)	37 (40)	Age	0.004
	Total	27 (34)	28 (33)	27 (31)	28 (33)	Interaction	1.000	37 (44)	32 (36)	29 (35)	32 (38)	Interaction	0.376
Soy	<65	33 (40)	29 (41)	29 (35)	30 (40)	Household income	0.641	28 (37)	35 (42)	35 (41)	34 (41)	Household income	0.109
	≥65	39 (47)	37 (39)	33 (36)	37 (40)	Age	0.055	42 (45)	41 (40)	42 (35)	42 (41)	Age	0.009
	Total	36 (44)	33 (40)	30 (35)	33 (40)	Interaction	0.884	37 (43)	37 (41)	37 (40)	37 (41)	Interaction	0.606
Vegetables	<65	138 (99)	132 (77)	144 (77)	136 (80)	Household income	0.393	152 (96)	157 (92)	166 (89)	159 (92)	Household income	0.275
	≥65	149 (106)	168 (84)	166 (86)	163 (90)	Age	<0.001	197 (104)	193 (102)	189 (99)	194 (103)	Age	<0.001
	Total	144 (103)	149 (82)	151 (80)	149 (86)	Interaction	0.169	181 (103)	171 (98)	170 (91)	173 (98)	Interaction	0.851
Fruit	<65	40 (53)	36 (52)	36 (51)	36 (52)	Household income	0.291	70 (82)	66 (72)	60 (71)	65 (73)	Household income	0.921
	≥65	65 (63) [*b]	78 (66)	74 (63)	74 (65)	Age	<0.001	99 (77)	92 (75)	83 (74)	94 (76)	Age	<0.001
	Total	54 (60)	56 (63)	47 (57)	54 (61)	Interaction	0.193	89 (80)	76 (74)	64 (72)	77 (76)	Interaction	0.863
Fish	<65	40 (38)	40 (36)	33 (32)	38 (36)	Household income	0.948	47 (44)	40 (38)	38 (35)	41 (38)	Household income	0.617
	≥65	55 (40)	50 (38)	53 (43)	51 (39)	Age	<0.001	50 (38)	47 (44)	45 (42)	48 (42)	Age	0.189
	Total	49 (40)	45 (38)	39 (37)	44 (38)	Interaction	0.164	49 (40)	43 (40)	40 (37)	43 (40)	Interaction	0.410
Meat	<65	35 (31) [*b,c]	47 (35)	53 (35)	47 (35)	Household income	0.002	37 (32)	45 (36)	46 (32)	44 (35)	Household income	0.049
	≥65	29 (32)	29 (24)	37 (31)	30 (27)	Age	<0.001	28 (27)	32 (28)	38 (29)	31 (28)	Age	<0.001
	Total	32 (32) [*c]	38 (31) [*3]	48 (34)	39 (32)	Interaction	0.058	31 (29)	40 (34)	45 (31)	39 (33)	Interaction	0.539
Eggs	<65	17 (18)	18 (16)	17 (16)	18 (16)	Household income	0.697	20 (20)	20 (19)	19 (17)	20 (19)	Household income	0.682
	≥65	19 (19)	17 (16)	20 (17)	18 (17)	Age	0.390	18 (21)	20 (20)	19 (16)	19 (20)	Age	0.480
	Total	18 (18)	17 (16)	18 (16)	18 (16)	Interaction	0.266	19 (21)	20 (19)	19 (17)	20 (19)	Interaction	0.569
Milk	<65	35 (48)	35 (52)	46 (63)	38 (55)	Household income	0.002	60 (71)	62 (72)	69 (73)	64 (72)	Household income	0.295
	≥65	38 (63) [*b,c]	56 (59)	58 (70)	52 (61)	Age	0.019	70 (78)	64 (68)	69 (63)	66 (71)	Age	0.820
	Total	37 (57) [*b,c]	45 (56)	49 (65)	44 (58)	Interaction	0.102	66 (76)	63 (70)	69 (71)	65 (72)	Interaction	0.850
Alcohol	<65	99 (159)	100 (165)	123 (180)	106 (168)	Household income	0.379	33 (122)	28 (82)	39 (102)	32 (94)	Household income	0.655
	≥65	82 (143)	94 (138)	83 (118)	90 (136)	Age	0.028	13 (61)	17 (54)	17 (54)	16 (57)	Age	0.001
	Total	89 (150)	97 (152)	112 (165)	99 (155)	Interaction	0.477	21 (89)	24 (73)	35 (96)	26 (82)	Interaction	0.575

[a] Means (SD) (g/1,000 kcal)

[*b] Multiple comparisons compared with 2–6 million yen, $p < 0.05$

[*c] Multiple comparisons compared with ≥6 million yen, $p < 0.05$

[d] Main effect or interaction by ANOVA, adjusted by household size
